# Supplementary material for: Experiences of children’s self-wetting (including urinary incontinence) in Cox’s Bazar’s Rohingya refugee camps, Bangladesh
Source: PLOS Glob Public Health. 2024 Mar 7;4(3):e0002362. doi: 10.1371/journal.pgph.0002362 (PMC10919867; doi:10.1371/journal.pgph.0002362)
Supplement: S1 Text — (DOCX) [file pgph.0002362.s002.docx]

# S1 Supporting Information: Extracts from the PhD thesis of C. Rosato-Scott detailing development of the Story Book Methodology

Using a rights-based approach, the starting-point from which to decide whether children should participate in any research project is that children have a right to be heard, even in a situation of crisis or its aftermath (1,2). From an initial stance of ‘involving children in research is the right thing to do’, researchers must decide if – for their particular project – it shouldn’t be done because a) the matter being researched doesn’t concern the child participants directly or indirectly; b) the researchers lack the capacity to either conduct the research or act on the findings; and/or c) the research could not be conducted ethically. When planning the research activities to be undertaken in Adjumani District and Cox’s Bazar, the Research Team considered these questions in turn:

a) Is the research of relevance to the children?

The Research Team concluded that the subject matter of the research (that is, the experiences of children living with incontinence) was directly of concern to the proposed child participants, and that only they – and not an adult proxy – could genuinely voice their experiences.

b) Does the Research Team have the capacity to conduct the research and act on the findings?

Of primary concern when involving children in research is to ensure that all necessary steps will be taken to safeguard the participants (3).This requires having researchers in the team with the minimum knowledge, skills and attitudes needed to facilitate and support meaningful participation with children (4 p.21). There is no shared standard for assessing a researcher’s competencies and capacities; instead assessment is subjective and context-specific (5). Guidelines such as (4 p.21) that provide ideal researcher specifications are therefore invaluable when recruiting those that will conduct the research. In Bangladesh the focus group facilitators were hygiene officers used to working with children. In Uganda the facilitators were research assistants from the Plan International Uganda database, known to have experience in qualitative data collection and who were familiar with the local community. The skills and experiences of the data collectors therefore met O’Kane’s minimum requirements including knowledge of local context; facilitation skills; and having an attitude that valued children (4 p.21). The participation of Plan International UK, Plan International Uganda, UNICEF Bangladesh and World Vision Bangladesh was viewed as a demonstration of an organisational mandate to a) learn more about how to best support people living with incontinence in a humanitarian context, and b) to incorporate the findings into their existing WASH programmes.

c) Can the research be conducted ethically?

As effective methodology and ethics go hand in hand, determining how the children would participate was crucial to deciding if the research could be conducted ethically (6). In late-2019, UK-based members of the Research Team met to initially design the research tools to be used. Virtual workshops (rather than in-person due to Covid-19) were then held in July 2020 with Australia-, Uganda- and UK-based members of the Research Team to contextualise the research methodology and individual tools to be used in Adjumani District; and in December 2020, with Australia- and Bangladesh-based members of the Research Team to contextualise the research methodology and individual tools to be used in Cox’s Bazar.

The research methodology was developed with the competencies and capacities of the proposed data collectors in mind. The day-to-day work of the proposed data collectors included focus group discussions (FGDs) and interviews with children aged five to 11, therefore the first decision point was whether to conduct FGDs or interviews. The Research Team’s preference was to avoid conducting interviews with young children to avoid any repercussions should a participant be viewed as having been specifically selected to take part in a conversation about such a highly personal, sensitive and often stigmatised medical condition (UI). It was also felt that an interview on such a topic could be an intimidating process for a young child with little benefit for them.

In contrast, FGDs are “purposeful, facilitated discussions between a group of participants with similar characteristics” (7 p.5). They generate data through interaction amongst the participants; and compared to an interview responses are deeper and more considered as participants have the opportunity to listen to others, reflect and consider their own viewpoint, and there is more scope for the natural emergence of issues (8). This is of course all reliant on the culture of the participants encouraging free expression and the Bangladesh- and Uganda-based members of the Research Team provided assurance that children in Cox’s Bazar and Adjumani District were able to express themselves in the context of a FGD without fear of punishment (5).

Agar et al. (3) found that FGDs work well with children, and they have also been shown to be an ideal qualitative research method when discussing sensitive topics with children: the group context can provide mutual support for shy children, articulate children can model for those lacking in confidence, and the peer support helps to redress the power imbalance that exists between adult and child during an interview (7,9). However, researchers need to balance the benefits of FGDs with the risks that a) disclosures may be shared outside of the group, and b) that discussion may stress or distress participants (3). Verbal introductions to FGDs must therefore outline that although the children can discuss the FGD with non-participants, details including who said what should not be shared, however there is a risk that they may be (3). The FGD facilitator must also emphasise that participation is voluntary, and anyone can leave at any time for any reason including if they do not want to speak or hear what is being said. Having a second person present to observe can also support the facilitator to recognise signs of stress or distress in participants, and take the appropriate action (10). The Research Team therefore concluded that conducting FGDs would be appropriate.

The size and composition of a focus group is critical in shaping the group dynamic (8). Members of the Advisory Team guided that FGDs with children should have up to six participants, which is in-line with the literature: (8) found that children are likely to feel more comfortable in a smaller group. Given the personal and sensitive nature of the issues being discussed, it was felt that groups should be split by gender, and it was also decided that groups should be split by age. This was partly due to the knowledge that the global prevalence of UI follows a trend of decline by age and therefore different age groups may have different experiences of the condition; but also following guidance from the Bangladesh Research Team who felt that children aged eight or more were noticeably more mature. The split was decided as five to seven-years old and eight to 11 years old. Further, the Bangladesh Research Team advised that the facilitator of the FGD should be of the same gender as the participants, particularly for the older ages (eight to 11) as otherwise they may be too embarrassed to contribute.

The research methods used during FGDs should reflect the capacities of the participants and ideally provide an opportunity for recreation and self-expression, particularly in a humanitarian context where such opportunities could be rare and therefore even more valuable (11). The use of drawing methods in research with children is known to be very successful as they can minimise the power relationship between adult researchers and the children; give participants time to think about what they want to communicate; help discussions about more complicated, sensitive and abstract issues; uncover subconscious perspectives; provide learning opportunities; and be fun and relaxing (6,12,13). Indeed in Cox’s Bazar members of the Bangladesh Research Team were already adapting methods used by Clowns Without Borders to educate the children on topics such as hand-washing. These methods included singing songs, playing games, telling stories, and drawing pictures.

The Research Team therefore designed a collaborative ‘Story Book methodology’, whereby the children collectively drew on sheets (‘Drawing Sheets’ with outline images provided) to create a story about an imaginary child living in Adjumani District or Cox’s Bazar who sometimes wet themselves. The group explored the feelings of the imaginary child at different times during the day (for example, when playing with friends) and including when they wet themselves, and the feelings of the imaginary child’s caregiver after an episode of self-wetting. The group also explored a time period, for example a morning, in the life of the imaginary child to understand the wider impacts of an episode of self-wetting and to ask the children for suggestions as to how to mitigate such impacts. Note that the use of an imaginary child rather than asking participants to share personal experiences of incontinence reduced the risk of a) a child becoming distressed at being asked to share such experiences and b) a participant being identified by friends, family and the wider community as experiencing incontinence which may result in negative consequences due to the stigma associated with the condition. This ‘imaginary’ approach was particularly favoured by the Bangladesh Research Team as some members knew of children that had participated in FGDs on menstrual hygiene management and who were later teased by fellow participants.

Given the age of the participants the Research Team agreed that the provision of compensation should be limited to the food and drinks provided during the FGD. And finally, (14) advise that methods and tools used in FGDs should be “informed by discussions with the children themselves and with adult community members” (p.20), and so the tools were modified after each FGD, informed by the children themselves. For example, after the first pilot FGD additional breaks were added to the FGD to ensure that the attention of the children was kept.

## References

1. United Nations. The United Nations Convention on the Rights of the Child [Internet]. New York, United States; 1990. Available from: <https://www.unicef.org.uk/what-we-do/un-convention-child-rights/>
2. United Nations Committee on the Rights of the Child. General Comment No.12. on the Right of the Child to be Heard [Internet]. Geneva, Switzerland; 2009. Available from: https://www.refworld.org/docid/4ae562c52.html
3. Agar, M., Macdonald, J., Basch, C.E., Bertrand, J.T., Brown, J.E., Ward, V.M., Bogardus, E.S., Charlesworth, L.W., Rodwell, M.K., Clark, C.D., Clark, L., Marsh, G.W., Davis, M., Igoe, J., Stember, M., Doswell, W.M., Vandestienne, G., Elliott, R., Fischer, C.T., Rennie, D.L., Garley, D., Gallop, R., Johnston, N., Pipitone, J., Greenbaum, T.L., Heary, C., Hennessy, E., Hill, M., Laybourn, A., Borland, M., Houghton, S., Durkin, K., Carroll, A., Hunter, A.J., Chandler, G.E., Kidd, P., Townley, K., Cole, H., Mcknight, R., Piercy, L., Kisker, E.E., Klein, J.D., Forehand, B., Oliveri, J., Patterson, C.J., Kupersmidt, J.B., Strecher, V., Krueger, R.A., Levine, I.S., Zimmerman, J.D., Lewis, A., Maccoby, E.E., Macmullin, C., Odeh, J., Mauthner, M., Mayall, B., Mcdonald, W.J., Topper, G.E., Mcmahon, M., Patton, W., Merrick, E., Merton, R.K., Kendall, P.L., Morgan, D.L., Morningstar, M.E., Turnbull, A.P., Turnbull, H.R., Rinne, M.G., Smith, M.W., Spethmann, B., Stanton, B.F., Aronson, R., Borgatti, S., Galbraith, J., Feigelman, S., Turner, S., Vaughn, S., Schumm, J.S., Sinagub, J., Wight, D., Greene, S. and Hogan, D. Exploring Children’s Views through Focus Groups In: S. Greene and D. Hogan, eds. Researching Children’s Experience [Internet]. Sage; 2005. Available from: https://dx.doi.org/10.4135/9781849209823.
4. O’Kane C. Review of children’s participation in humanitarian programming [Internet]. London, United Kingdom; 2013. Available from: https://resourcecentre.savethechildren.net/document/review-childrens-participation-humanitarian-programming/
5. Bennouna, C., Mansourian, H. and Stark, L. Ethical considerations for children’s participation in data collection activities during humanitarian emergencies: A Delphi review. Conflict and Health. 2017;11(5).
6. Thomas, N.P. and O’Kane, C. 1998. The ethics of participatory research with children. Children & Society. 1998;12:336–348.
7. Lansdown, G. and O’Kane, C. A toolkit for monitoring and evaluating children’s participation. Booklet 3 [Internet]. 2014. Available from: https://resourcecentre.savethechildren.net/node/8080/pdf/me_toolkit_booklet_3_low_res1.pdf.
8. Finch, H. and Lewis, J. Focus Groups In: J. Ritchie and J. Lewis, eds. Qualitative Research Practice - A Guide for Social Science Students and Researchers. Sage, 2003;170–198.
9. Jones, L. Responding to the needs of children in crisis. International Review of Psychiatry. 2008;20(3):291–303.
10. Feinstein, C., Fyrk, K., Gezelius, H., Heiberg, T., Jareg, E., Karkara, R., Modig, C., Nilsson, M., Sheth, A. and Stevenson, S. So You Want to Involve Children in Research? [Internet]. 2004. Available from: https://www.savethechildren.org.uk/content/dam/global/reports/education-and-child-protection/so-you-want-to-involve-children-in-research.pdf.
11. Berman G, Hart J, O’Mathúna D, Mattellone E, Potts A, O’Kane C, et al. What We Know about Ethical Research Involving Children in Humanitarian Settings An overview of principles, the literature and case studies, Innocenti Working Paper No. 2016-18 [Internet]. Florence, Italy; 2016. Available from: https://www.unicef-irc.org/publications/849-what-we-know-about-ethical-research-involving-children-in-humanitarian-settings-an.html
12. Eldén, S. Inviting the messy: Drawing methods and ‘children’s voices’. Childhood. 2013;20(1):66–81.
13. Literat, I. ‘A pencil for your thoughts’: Participatory drawing as a visual research method with children and youth. The International Journal of Qualitative Methods. 2013;February.
14. Z`
