# Supplementary material for: Experiences of children’s self-wetting (including urinary incontinence) in Cox’s Bazar’s Rohingya refugee camps, Bangladesh
Source: PLOS Glob Public Health. 2024 Mar 7;4(3):e0002362. doi: 10.1371/journal.pgph.0002362 (PMC10919867; doi:10.1371/journal.pgph.0002362)
Supplement: S1 Table — (DOCX) [file pgph.0002362.s003.docx]

# **Supporting Information 3: Full codebook**

| **Thematic Area** | **Sub-Categories** | **Code** | **Explanation, examples** |
| --- | --- | --- | --- |
| Demographic Information | Detailed information about the research participants including Children, Caregiver, Community Members and NGO officials | 1. Children’s Age 2. Children’s Gender 3. Caregiver’s Age 4. Caregiver’s Gender 5. Relationship with children 6. Number of Family Members 7. Number of Children 8. Role of the NGO officials | 1. To identify the age of interviewed children 2. To identify the gender of interviewed children 3. To identify the age of the caregiver of the interviewed children 4. To identify the gender of the interviewed caregiver of the children with incontinence issue 5. To identify the relationship with children with incontinence of interviewed caregiver 6. To measure the number of family members of the children with incontinence 7. To identify the number of children of the family from 5 years old to 11 years’ old 8. To understand the role and designation of the NGO officials who are working in the Rohingya humanitarian settings and their relationship with the children |
| Issues of incontinence related to WASH | Children’s Perspective | Toilet usage | Understanding the issues of children to reach the toilet such as fear of using toilet at night, uncomfortable to use toilet, uncleaned toilets. |
|  |  | Support received | Identifying the support of caregivers/family members to help the children to access the toilet facilities |
|  |  | Struggles of children | Understanding the children’s experiences including facilities and struggle of toilet usage |
|  | Caregiver’s Perspectives | Available facilities | Identifying the available facilities (such as available of water, handwashing component) for children’s toilet usage from caregiver perspectives |
|  |  | Child-friendly toilet | Understanding whether the toilets are suitable for children usage (e.g. the location of the toilet, higher toilet base from the ground, the pedestrian of the toilet are not suitable for children usage) Identifying the placement or location (High place or Low Place) of the toilets to understand whether these are accessible for children |
|  |  | Reactions from community | Understanding whether the community members are helpful towards this incontinence issue of the children and their reactions towards incontinence |
|  |  | Seek for support | Understanding whether their children need any kind of support from the caregiver/ family members to use toilet facilities |
|  |  | caregiver’s perception of problems | Understanding the children’s experiences including facilities and struggle of toilet usage from caregiver perspective |
|  | Service Provider’s Perspectives | Multiple user | Identify the user of the toilets and the condition of using toilets |
|  |  | Service provider’s perception of facilities | Identifying the available facilities for children at the toilets from service providers perspectives |
|  |  | Service provider’s perception of problems | Understanding the toilet usage experience of children that the service providers encountered |
|  |  | Wash hygiene behavior | Understanding the knowledge, awareness, attitude and practice of children’s WASH hygiene behavior |
|  |  | Struggle to provide assistance | Understand the struggles that the service providers face to assist/helpthe caregivers and children with incontinence issue |
| Issues of incontinence related to Inclusion | Children’s Perspective | Support needed | Understanding the support ( Taking the children to the toilets) of the children which are need to be inclusive in WASH |
|  |  | Accessibility of children | Understanding the issues including facilities and problems the children face to access the WASH facilities |
|  |  | Availability for children | Understanding the availability (number of wash facilities, distance, location) of WASH for children in the case of incontinence issue of children |
|  | Caregiver’s Perspectives | ,  Caregivers perception of accessibility | Understanding the issues including facilities and problems the children face to access the WASH facilities from caregiver’s perspective |
|  |  | Caregivers perception of availability | Understanding the availability (number of wash facilities, distance, location) of WASH for children in the case of incontinence issue of children from caregiver’s perspectives |
|  |  | Caregivers perception of awareness | Understanding whether the caregivers/ family members are aware about toilet usage and incontinence |
|  |  | Social norms and regulation | Understanding whether the social norms, rules and regulations that are followed by the family members to address the incontinence issue of the children are inclusive or not |
|  | Service Provider’s Perspectives | Service providers perception of accessibility | Understanding the process and techniques of make the toilets accessible (child friendly) for the children |
|  |  | Service providers perception of availability | Ensuring the availability of wash facilities including toilets and hygiene products for the children |
|  |  | Service providers perception of awareness | Creating awareness among the community including community people and caregivers/ family members regarding the usage of wash facilities and incontinence |
| Issues of incontinence related to Protection | Children’s Perspective | Fear | Feeling of an unpleasant emotion caused by the threat of danger or harm while using wash facilities |
|  |  | Comfortability | being in a state of physical or mental comfort while using toilets |
|  |  | Safety | condition of being protected from or unlikely to cause danger, risk, or injury while using toilets |
|  | Caregiver’s Perspectives | Safety concern | Concerning of an unpleasant emotion caused by the threat of danger or harm while the children are using wash facilities |
|  | Service Provider’s Perspectives | Unpleasant experience | Stating an experience of an unpleasant incident caused by the threat of danger or harm while the children are using wash facilities |
| Facilities, resources, and services targeted to incontinence | Facilities, resources, and services targeted to incontinence from the Service provider’s perspective | Need Assessment of the children | A systematic process for determining and addressing needs of the children |
|  |  | Caregiver’s requirement | A systematic process for determining and addressing requirements of the caregiver for the children |
|  |  | Available facilities, resources, and services | Identify the available facilities, resources and services targeting children’s incontinence |
|  |  | Coordination | The different teams of an organization such as health, protection, children safety coordinated with one another to address the incontinence issue of the children. |
|  |  | Policies | Relevant policies targeting incontinence of the children’s caregiver |
|  |  | Service strategies & guidelines | Relevant service strategies and guidelines focusing on incontinence of the children’s caregiver such as awareness guidelines, household intervention |
| Experiences of incontinence | Children’s Experiences of incontinence | Reasons identified by children | Identifying the cause of an event or situation or something that provides an excuse or explanation related to incontinence |
|  |  | Struggle faced by children | Experience difficulty and make a very great effort in order to do something by the children with incontinence |
|  |  | Awareness of children | Being knowledgeable and conscious about the incontinence of the children |
|  |  | Reactions of children | Something done, felt, or thought in response to incontinence by the children |
|  |  | Self-Management | The process of dealing with or controlling things or people related to incontinence by the children themselves |
|  | Caregiver’s experiences of incontinence | Reasons identified by caregivers | Identifying the cause of an event or situation or something that provides an excuse or explanation related to incontinence by the caregivers |
|  |  | Struggles faced by caregivers | Experience difficulty and make a very great effort in order to manage incontinence of the children |
|  |  | Awareness of caregivers | Being knowledgeable and conscious about the incontinence of the children |
|  |  | Reactions of caregivers | Something done, felt, or thought in response to incontinence of the children |
|  |  | Management strategies by caregivers | The process of dealing with or controlling things or people related to incontinence by the caregivers |
|  | Service provider’s experiences of Incontinence | Reasons identified by service providers | Identifying the cause of an event or situation or something that provides an excuse or explanation related to incontinence by the service providers |
|  |  | Struggle identified by service providers | Experience difficulty and make a very great effort in order to manage incontinence of the children |
|  |  | Awareness program | Programs are held to make others knowledgeable and conscious about the incontinence of the children |
|  |  | Management strategies of service providers | The process of dealing with or controlling things or people related to incontinence |
| Attitudes towards incontinence | Children’s attitude towards incontinence | Scared | being in a state of fear, fright, or panic of punishment because of incontinence issues at home or school or community |
|  |  | Tensed | Feeling very nervous and worried and unable to relax because of facing incontinence issue regularly at school/home/community |
|  |  | Sad | Feeling or showing sorrow; unhappy because of facing unwanted situation regarding incontinence issue |
|  | Caregiver’s attitude towards incontinence | Discomfort | Feeling uncomfortable, uneasy to deal with children’s incontinence issue |
|  |  | Feeling embarrassed | Feeling embarrassed or feeling ashamed of, sharing the children’s incontinence issue with others |
|  |  | Feeling bad/upset | Saddened or disappointed about incontinence issue of the children |
|  | Community People’s attitude towards incontinence | Annoyed | Feeling angry over the incontinence issue of the children |
|  |  | Supportive | Showing encouragement and provide guidance or suggestion to the caregivers regarding children’s incontinence issue |
| Improvement of Emergency WASH programming | Available WASH Services | Child-friendly Toilet | Focusing on the toilets which are suitable for the children to use |
|  |  | Toilets in reach | The toilets are easier to access for the children |
|  |  | Increase WASH facilities | Wash facilities includes water, hand washing facilities and others |
|  |  | Ensure protection using toilets | Protection focuses on the safety and security of the children while using toilets |
|  | Scope of Improvement | Ensure availability of WASH services | Availability includes the water, handwashing station, handwashing product and others |
|  |  | Ensure accessibility into WASH services | Understanding the process and techniques of make the toilets accessible (child friendly) for the children |
|  |  | Community feedback | Addressing the feedback from the community members including caregivers and others to develop the emergency WASH programming for the children |
| Improvement of Emergency Protection programing | Available protection services | Safety | condition of being protected from or unlikely to cause danger, risk, or injury while using toilets |
|  | Scope of Improvement | Ensure availability of protection | Availability of protection facilities which includes safety, security and protection from harm while the children are using toilets |
|  |  | Ensure accessibility of protection services | Understanding the process and techniques of make the toilets safer (child friendly) for the children |
|  |  | Protection service strategy | Understanding the strategies of the protection services; such as, in what cases or in which situation or for whom or in which process the protection service will be provided |
