# Supplementary material for: Experiences of children’s self-wetting (including urinary incontinence) in Cox’s Bazar’s Rohingya refugee camps, Bangladesh
Source: PLOS Glob Public Health. 2024 Mar 7;4(3):e0002362. doi: 10.1371/journal.pgph.0002362 (PMC10919867; doi:10.1371/journal.pgph.0002362)
Supplement: Attachment — Submitted filename: Alam et al RTR.pdf [file pgph.0002362.s005.pdf]

THE UNIVERSITY OF WESTERN AUSTRALIA

Dr Dani Barrington
Senior Lecturer in Global Hea;th
School of Population and Global Health

Dr Sharon Alane Abramowitz
Academic Editor
PLOS Global Public Health

16 November 2023

Dear Dr Abramowitz

RE: Reviewer Response, PGPH-D-23-01619: Experiences of children's self-wetting (including urinary incontinence) in Cox's Bazar's Rohingya refugee camps, Bangladesh

Many thanks for allowing us the opportunity to revise our manuscript based on the comments provided by the two reviewers. We have addressed each of their comments and provide an explanation for each change in the letter below.

We believe that the manuscript is ready for publication in PLOS Global Public Health, but are happy to make further revisions if you or the reviewers deem them necessary.

Yours sincerely

Dr Dani Barrington
On behalf of co-authors

Additional Editor Comments (if provided):

As you will see from the included individual reviews and comments, both reviewers found that there were substantial contributions to knowledge for humanitarian settings. Each reviewer made a different recommendation for publication, and both made recommendations that can seem contradictory. Having reviewed the manuscript and Reviewer 1 and Reviewer 2's feedback, I believe that the manuscript has significant merit and can be made ready for publication with major revisions. In particular, we recommend that the researchers address Reviewer 1's comments, particularly (a) providing additional information about the research team, and (b) the last paragraph, which calls for greater data clarity and a discussion of policy implications. We recommend that you pay careful attention to the totality of Reviewer 2's comments, which have substantive insights for defining the problem, providing context, clarifying data, etc. Please note that you should position your manuscript towards the audience that you feel is most appropriate.

Many thanks for your consideration of the reviewers' comments in their totality. The manuscript was always meant to focus on how WASH and protection practitioners in humanitarian settings can contribute to improving the experiences of children who self-wet; we did not investigate medical causes or treatments for urinary incontinence, and we have thus revised the manuscript to be clearer that such specialists (academics and practitioners) are the intended audience. By being clearer that we did not investigate incontinence or its prevention/treatment through a medical lens, we believe we have addressed many of Reviewer 2's concerns.

We have also revised the manuscript to provide further details on the research team and implications for policy and practice (this is detailed more in the Discussion than the Conclusion), being clear that this was only a small study within two camps of a single humanitarian situation. We detail how we have made those revisions in our response to individual reviewer comments, below.

Reviewer #1:

The manuscript appears to be technically sound, and the data collection methods used are well-described. The authors conducted interviews and Story Book sessions, which allowed them to gather a range of perspectives from children, caregivers, and other community members in Rohingya refugee camps. Ethical considerations have been appropriately discussed, indicating a rigorous approach to research ethics. However, I would like to see a small section on the epistemology of the research and background discussions as to why this perspective was necessary in conducting this field work. Also, whether the fidelity to original epistemology was maintained throughout or was subjected to change due to some unanticipated stimuli. Please explain.

Deciding to conduct research with extremely vulnerable children, and the method by which to do so, was a long process, and difficult to restrict to a short section of a journal article. However, we understand Reviewer 1's interest in this and why it may be of concern to other readers. As such, we have added a new piece of Supporting Information to our submission, extracts from the PhD thesis of co-author Claire Rosato-Scott, which detail the literature we interrogated to inform our decision to undertake work on this topic with children, and develop the Story Book research method. We have included a new, short paragraph in the manuscript which refers to the Supporting Information:

> *"Self-wetting is a sensitive topic; the research team had to consider carefully whether it was appropriate to include children as participants in such a study. The process of consideration and development of a methodology that was appropriate for vulnerable children are detailed in Supporting Information 1. Ultimately, we decided to include children as they have a right to be heard in research which concerns their personal experiences, and because across the research team we had the necessary expertise to conduct the work in a sensitive and protective manner."*

Please also add a positionality statement that discusses how the researchers mitigated unconscious bias and microaggression with sub-sections on reflexivity.

Additionally, the paper should also present a debate on Etic Vs. Emic perspectives, given that all the researchers in the camps were outsiders. How did camp residents view the researchers? What challenges did this pose? How were these challenges mitigated?

We have added a section on positionality and reflexivity to the Methods section:

> *"Data was collected by individuals who do not reside within the camps, but who were familiar to the participants through their work for WVB-CXB. This was appropriate as participants were likely more open to discussing such a sensitive topic with outsiders in*

their professional capacity, as there would be less chance of negative repercussions from disclosure, than if data collectors had been recruited from within the camps. Children and their caregivers appeared comfortable with data collectors and spoke openly about the challenges they face regarding self-wetting.

We endeavoured to uncover both emic and etic perspectives through the research methods. An etic approach allowed us to understand how the experiences of children and caregivers in this specific context may be similar to other humanitarian contexts and human experiences of self-wetting generally, and an emic approach allowed us to interrogate how self-wetting is experienced within the local culture and religion of the camps (a crucial consideration given that societal shame is a common aspect of self-wetting experiences). Using this mixed approach, we could identify aspects of the self-wetting experience which are likely context specific and those which may be universal, as well as practical measures which may be taken by humanitarian practitioners to improve experiences in this specific context, or which may be applicable more generally.

Data collectors did not take formal fieldnotes, but at the end of each day of data collection, the research team conducted a debrief session to critically reflect on the process and adjust the methods as necessary, for example, potential probes for SB sessions and interviews that could be used to identify and explore emerging themes. The team also discussed any verbal, nonverbal, and environmental slights, snubs, or insults, whether intentional or unintentional, which data collectors may be using, which may be communicating negative messages about those who self-wet to children or their caregivers. The team members then discussed how to avoid those unintentional biases and microaggressions and put this into practice in subsequent days."

The study can also benefit from usage of a theoretical framework. Please revisit your results and see if these findings can be linked to potential theories (embodiment/minority stress/rational choice theory etc.).

We have carefully considered how useful it would be to present our findings using a theoretical framework. We believe that given the practical focus of this paper for WASH and protection humanitarian scholars and practitioners (who we believe are now more clearly our target audience), and its potential publication in a public health, rather than social science, journal, the use of a theoretical framework may draw the attention of our intended audience from the raw experiences and practical implications of the work.

The data presented in the manuscript appear to support the conclusions drawn by the authors. The findings, including the negative physical, social, and mental health impacts of self-wetting on children and caregivers, are well-supported by the qualitative data collected during the study. Participants provided insights into the causes and consequences of self-wetting, as well as suggestions for potential solutions.

Thank you for this comment.

If this study has made use of data enumerators from the local region (who are not authors), please acknowledge them one-by-one.

We have added the names of research trainers and data collectors to the acknowledgements.

However, the article could benefit from a more structured presentation of the data, potentially using thematic tables or figures to enhance clarity and facilitate a more straightforward understanding of the key findings. Please make use of visuals.

Thank you for this suggestion. We have summarised the quantitative data in each Results section into tables (Tables 2, 3 and 4) and removed it from the text. We believe this makes the findings easier to digest and removes the distraction of numbers from the core text, instead focusing the text on illustrative examples from the qualitative data. The full codebook of the study is included as Supporting Information 3.

We believe the figures of the children's drawings and our proposed model (Figures 1, 2 and 3) suffice as graphics to represent our findings. We have added a fourth figure showing the educational poster we developed as an output of this research study.

Additionally, while the data support the conclusions, the discussion section could further explore the implications of the findings for policy and practice in refugee settings, providing a stronger link between the research and potential interventions.

We now include in the Discussion section details of ongoing work in the WASH sector to roll out education and practical interventions to improve the experiences of those who self-wet in humanitarian situations globally:

> *"As a result of this research project, we have developed a contextually appropriate, editable poster that can be displayed in shared public spaces (e.g., child-friendly spaces in the Rohingya camps), explaining that most children will grow out of self-wetting, and should be supported in the meantime (Figure 4). The poster was designed to represent Rohingya individuals but can be used in other contexts where the depictions of individuals are considered appropriate.*
>
> *Figure 4: English version of poster designed to educate the public that most children will grow out of self-wetting and should be supported. The posters are editable, so the text can be changed to any language, and other text can be added as necessary. The white space at the bottom is for local organisations to add their own contact details if viewers would like to know about self-wetting. An editable version of the poster is available from [25] (*www.doi.org/10.17605/OSF.IO/E3KHV*).*
>
> *Humanitarian professionals across the globe have called for further knowledge and training on how to address continence issues in emergency contexts [17]. Since the completion of this project, we have worked with Oxfam and the Research Grants Institute of Ghana to develop a training package for humanitarian practitioners based on our combined research on self-wetting in Bangladesh, Ethiopia, Malawi, Uganda and Ghana, as well as existing literature. The training package educates humanitarian practitioners on self-wetting and how simple WASH and protection interventions may assist in improving the experiences of people who self-wet of all ages, and their caregivers [REF]. This training package is currently being trialled by Oxfam in 11 different humanitarian situations to understand its applicability in a range of contexts.*

Reviewer #2:

Thank you for the opportunity to review this paper. I was really pleased to see this issue explored in a study – especially as it is so underrecognized by humanitarian actors and there is a lack of evidence on the drivers or solutions in humanitarian settings.

That being said, this paper needs a significant amount of work to address one or some of these gaps effectively. It may be more effective to rework the paper and direct it very clearly and specifically at a WASH audience. The current draft is heavily focused on WASH aspects of incontinence, so reworking it in this way would require a less extensive revision.

The manuscript was always meant to focus on how WASH and protection scholars and practitioners in humanitarian settings can contribute to improving the experiences of children

who self-wet; we did not investigate medical causes or treatments for urinary incontinence. We understand that this was unclear in our initial submission. We have thus revised the manuscript to be clearer that such scholars and practitioners are the intended audience, and that we do not mean to imply that they are qualified to prevent or treat the medical condition of urinary incontinence. For example, we now say in the Abstract:

> *"We particularly focused on how water, sanitation and hygiene (WASH) and protection interventions might assist in improving these experiences and reducing negative attitudes towards those who self-wet...*
>
> *"… Medical intervention for UI is likely necessary to improve experiences in some cases, but that was beyond the scope of this study and the qualifications of WASH and protection practitioners. However, although WASH and protection practitioners are unable to drastically alter camp conditions (physically, but also with regards the general trauma of displacement, which is a known contributor to UI) or treat UI, the lives of children who self-wet in these camps could likely be improved by increasing awareness on self-wetting to decrease stigma and ease the concerns of caregivers, as well as increasing the number of toilets, ensuring they are well-lit, providing child-friendly toilets and cubicles, fixing the roads/paths that lead to sanitation to facilities and increasing the provision of continence management materials."*

We have also made other changes throughout the manuscript, for example, being clearer that we did not investigate urinary incontinence from a medical perspective, but only the symptom of self-wetting, which may also be caused by inappropriate WASH facilities. For example:

> *"Throughout this manuscript, we use the term 'self-wetting' when a child experiences urine leakage, regardless of whether this is UI or SUI (as both of these appeared to be occurring in the study population, and although we asked participants what they believe causes urine leakage, our methods did not allow for medical diagnoses)."*

and

> *"Although WASH and protection practitioners do not have the qualifications to prevent or treat the medical condition of UI, there are three 'pain points' in the model where we believe these practitioners can remove or reduce factors which contribute to poor well-being, thus improving the experiences of children who self-wet and their caregivers. These are improving WASH facilities, providing continence management supplies and increasing knowledge of self-wetting whilst reducing stigma."*

I was surprised to read that the introduction doesn't even mention the role of adversity – especially in humanitarian settings – and hardly mentions the psychological drivers of self-wetting. There are two words in a sentence on page 3 line 74 ("situation-induced trauma, anxiety…" that is in specific reference to humanitarian settings. Otherwise, the paper only refers to physiological and social causes. Incontinence is not a binary issue but is very complex and frequently has psychological drivers and nearly always has psychological consequences and exacerbating factors. This is true for settings experiencing crisis as well as all other settings.

We agree that there are psychological drivers of UI, which was not clear before. We have added a note in brackets to indicate that 'health' does not just refer to physiology, and added 'psychological' alongside 'physiological' later in the Introduction:

> *"The medical condition of urinary incontinence (UI) is defined as the involuntary loss of urine, and it is a symptom of a range of health issues (UI has a range of physical, mental and emotional causes) [1]. It is a complex global health issue that has a negative impact on people's security, dignity, rights and general quality of life [2, 3]. In addition to UI, sometimes people 'wet' themselves because they do not want to, or cannot,*

> *access a toileting facility in time; not due to a physiological or psychological inability to stop urine leakage."*

As Reviewer 2 notes, we do only briefly state that trauma, particularly in humanitarian contexts, can drive UI. As the paper is directed towards a WASH/protection, rather than medical, audience and these practitioners are not qualified to prevent or treat the medical condition of incontinence, we do not believe it is useful to add more text on trauma-induced self-wetting, but to focus more on practical WASH and protection management strategies.

The general living conditions for everyone in the camp – crowding, very basic shelters, fire risk, flooding risk, prevalent interpersonal violence, experiences of caregivers and often of children to torture (witnessing it, surviving it) and extreme violence and subsequent psychological distress or morbidity – these are exceptionally severe conditions. The latrine situation compounds this and has a huge impact on quality of life as well as safety – but the authors focus almost exclusively on the WASH situation rather than the life situation, and they don't consider how adversity and the responses of different people (parents and teachers beating or shaming children, for example) influence the prevalence of self-wetting, type of self-wetting, experience of it, and children's as well as adults' responses to it. This is a major weakness in this paper. The methods are insufficiently described to determine whether or not the study can capture this. However, the authors could triangulate what is known about the bio-psycho-social aetiology of enuresis and encopresis.

Thank you for this comment; we agree that there are a myriad of factors contributing to experiences of self-wetting in this context. Unfortunately, we're not in a position to be able to comment on the impact of adversity or causation from such a small cross-sectional pilot study in two camps of Cox's Bazar. The impact of adversity on a child's experience of incontinence/adult responses to it is interesting; we know much about both in non-humanitarian settings, and we are now trying to know more about the impact in humanitarian settings. Given the limitations of our methods (as discussed in the manuscript, we did not purposively recruit for children who self-wet, and the children we did speak with told us about the experiences of a fictional character, not necessarily themselves) and small study size, we have chosen to focus our findings on how WASH/protection interventions may improve wellbeing related to self-wetting, knowing that such interventions alone will not address all of the issues associated with self-wetting causes and experiences. We hope this is now clearer in the manuscript with our framing for a WASH/protection audience.

Self-wetting seems to be the focus of this paper yet the authors provide evidence on faecal incontinence – which also has huge psychological and social drivers and also different solutions. The term used, "self-wetting" appears to refer to both urinary and faecal incontinence, but this is never made clear. The paper would be improved if the authors explored the findings (if available) for nocturnal enuresis, diurnal enuresis, encopresis (faecal incontinence) and a combination of enuresis and encopresis. Right now it isn't clear when the authors refer to self-wetting what they refer to. If the study methods don't allow for the above suggestion, this should be clarified in the text.

Apologies that this is unclear, our study always set out to focus on urine leakage, but some participants chose to speak about faecal leakage as well. We have re-written the Introduction to be clearer that we are focusing on urine, and removed all mentions of faeces except where they are integrated in illustrative quotes from participants:

> *"The medical condition of urinary incontinence (UI) is defined as the involuntary loss of urine, and it is a symptom of a range of health issues (UI has a range of physical, mental and emotional causes) [1].It is a complex global health issue that has a negative impact on people's security, dignity, rights and general quality of life [2, 3]. In addition to UI, sometimes people 'wet' themselves because they do not want to, or cannot, access a toileting facility in time; not due to a physiological or psychological inability to*

stop urine leakage. This is sometimes known as socialurinary incontinence (SUI)… Children aged between five and 11 years occasionally wet themselves as a result of the medical condition of UI. and can also experience SUI. Throughout this manuscript, we use the term 'self-wetting' when a child experiences urine leakage, regardless of whether this is UI or SUI (as both of these appeared to be occurring in the study population, and although we asked participants what they believe causes urine leakage, our methods did not allow for medical diagnoses)."

We have also added 'urinary' to the paper's title.

The study also found some instances where parents report beating their children and then feeling sad – it is not what makes the parent sad, however the authors appear to attribute the sadness to the child's incontinence. Another interpretation of the exact quote lines 344-346 is that the mother feels sad about beating the child and about the child's incontinence. This quote is a very powerful one, and it captures very well the complexity of the issue – children not able to control their bladder or bowels, a parent's response, a parent's emotions in the moment and afterwards on reflection. The current analysis doesn't unpack this, and the result is a valid but oversimplified recommendation for a solution.

The authors have developed a causal model for their findings but the methods cannot support such a model. This research can only inform a theoretical model which requires testing.

We agree that in the illustrative quote provided it could be interpreted that the mother is sad because the child self-wet, and/or because they beat the child. However, our finding, "One of the main reasons their heroes were scared is that they believed their (heroes') mother would scold them because she was upset, furious, sad and/or embarrassed" was based on the responses of children across 7 of the 8 SB sessions, not this individual quote. Our analysis of the data indicates that children, in general, believed their mothers were 'sad' because they had self-wet, not because of the punishment. That being said, a third person (the child) cannot be expected to accurately attribute a mother's feelings to a specific cause, so it may well be that the mother is sad about the beating (or both the beating and the self-wetting). We do not attempt in the manuscript to ascribe causality to the mothers' negative feelings, but merely to indicate that they are clearly experiencing negative emotions relating to their child self-wetting. Such negative emotions are likely influenced by a myriad of things (for example, the ongoing trauma of a humanitarian setting), and our methods did not allow us to unpack these beyond investigating simple ways by which WASH and protection programming improvements might assist in reducing negative experiences. We agree that it would be valuable for future work to unpack more fully how negative emotions related to self-wetting are influenced by a myriad of environmental and social factors. We have altered the Discussion section to be clearer that the model we have developed is based only on the data collected in this study, and does not encompass the full complexity of the situation:

"As discussed in the Introduction, there are a range of physiological and psychological causes of self-wetting, and experiences of self-wetting will be influenced by the physical, social and cultural environment a child and their caregiver reside within, as well as individual biological factors. In this small study we asked participants to provide details of their experiences, as well as their insights on the causes of these experiences and their eventual impacts on wellbeing. This likely did not encompass the entire reality of how life within an emergency context affects self-wetting. However, based upon the responses of participants we have developed a theoretical model that indicates some of the factors which contribute to negative experiences and wellbeing impacts (Figure 3). Although WASH and protection practitioners do not have the qualifications to prevent or treat the medical conditions of UI, there are three 'pain points' in the model where we believe these practitioners can remove or reduce factors which contribute to poor well-being, thus improving the experiences of children who self-wet and their caregivers. These are improving WASH facilities, providing continence management supplies and

increasing knowledge of self-wetting whilst reducing stigma. The model could likely be expanded to include more factors which contribute to negative experiences and impacts as further studies are conducted, providing further guidance on how experiences of children and caregivers could be improved."

The discussion mentions some important consequences of the self-wetting – most notably experiencing multiple forms of violence, withdrawal or absence from school and social activities – but they stop short of describing what this can mean for children's life trajectories. This is a critical weakness of the paper – exploring this could provide a far stronger argument for improving the WASH conditions for children in the camps.

Thank you for this comment, we agree that attending school and social activities will likely have a positive influence on life trajectories of children, but we believe it is beyond the scope of this paper to need to convince readers of why participating in such activities is beneficial.

The focus on self-wetting and solutions for self-wetting in the conclusions in both the text and in the abstract imply that self-wetting is an isolated problem for children. The self-wetting is clearly related to lack of adequate WASH facilities but it is very likely to also be related to experiences of adversity, some of which the authors have described, as well as additional types of severe adversity. The references to encopresis lends further strength to the role of adversity in children's incontinence in the study sites. Improving latrines would make a huge difference to these children, their families and their communities – but it is unlikely to solve the problem for most children.

We believe we have addressed this comment by more clearly defining self-wetting and orienting the paper to a non-medical audience (extracts from the revised manuscript inserted above). There is certainly the need for medical intervention to treat UI for some children in the camps, but we did not investigate this.

Last but not least, the issues presented here – lack of access to adequate sanitation facilities for children, limited access to water, physical abuse, psychological abuse, exclusion from school – these are child rights violations. The way self-wetting is described here suggests that it is very widespread in the study sites. If so, this should be clarified (what is the prevalence? If not known, did the community think self-wetting is common?). The self-wetting is an example of a real, profound impact of these child rights violations not only on the children but on the caregivers and the community. This study lends valuable evidence to support why we should, and must, enable the conditions for children to realise their basic rights.

As stated in the paper, it is very difficult to determine the prevalence of self-wetting in any context, particularly low resource settings; "*To the best of our knowledge, the prevalence of childhood self-wetting, including UI, has not been successfully measured in low resource settings, with a recent study that attempted to do so noting that the stigma associated with self-wetting makes it a challenging task to identify children who experience it* [18]." At the time of this study, no one had previously quantified self-wetting in humanitarian contexts (some work was undertaken by our colleagues at Oxfam at the same time as our study, and they may now have prevalence data for selected sites in Ethiopia and Malawi).

However, humanitarian practitioners believe self-wetting is common in emergency settings, and our study was funded under a call to understand better what is happening in these contexts. We elected to conduct a smaller, in-depth qualitative study to understand experiences, rather than a quantitative study to understand prevalence. As such, the prevalence of self-wetting by children in CXB remains unknown, although our study does suggest it is not uncommon, and this is stated in the original paper submission, "*Participants indicated that the Rohingya community usually refer to self-wetting as "Korai", and it is common among children aged five to 11 years."*

This paper would be more effective if it were specifically limited to a WASH sector audience – and this should be clearly stated at the beginning and in the abstract. The discussion section on medical interventions should be deleted – it lacks nuance and it is not the focus of the paper or the study, based on what is presented.

We have re-focussed the paper to be intended for WASH/protection audiences; excerpts from the manuscript are included above. We have removed the section on medical intervention.

Other comments:

Page 3 lines 50-51 – this is a sweeping generalization backed by an outdated reference that is focused on adults including people with physiological incontinence and the elderly. This reference is not relevant for children, especially not for children in humanitarian settings. Please provide sound evidence to back this claim.

This paragraph is not specifically about children, but people who self-wet in general. However, we have now removed this reference and replaced it with a recent citation.

Page 3 Line 53 – this is incorrect. Enuresis refers to self-wetting. Wetting during sleep is called nocturnal enuresis. Daytime wetting is called diurnal enuresis, as it usually also occurs during sleep.

We used International Children's Continence Society definitions; they no longer use the term diurnal enuresis (as per the reference). However, we have removed the term enuresis entirely from the revised manuscript as it was an unnecessary medical term for our target audience.

It's great that the authors considered feeding back to the communities, and it is unfortunate that COVID-19 disrupted the plans for this. I am well aware that the restrictions in access to the CXB camps have been particularly challenging, however access has markedly improved during the past couple of years and it is still worth going back to the communities and at the very least sharing the findings, if not internally validating them. This is particularly important for research on sensitive topics with particularly marginalized populations, both for accountability to the population and also to foster trust in the genuine goodwill of researchers. World Vision still has a presence in at least some of the camps. Why were the feedback sessions not adapted to overcome COVID-19 access limitations?

It is incredibly difficult for us to conduct feedback sessions without extra funding. During the project period, World Vision Bangladesh-CXB provided their time in kind for the data collection, as part of a contract with UNICEF. World Vision Bangladesh-CXB are no longer able to provide their time in-kind and we do not have any funding for their time. It would be inappropriate (and likely impossible with regards permits/approvals) for us to share the findings ourselves. However, we have provided World Vision Bangladesh-CXB with the poster that is discussed in the revised manuscript, and they have indicated that they will be able to share copies of this in the camps when conducting their regular visits.

The method of data collection is not even briefly described and the reader is simply given a reference so they can go look it up if they're interested. Later in the results, children talk about heroes wetting themselves – which will sound very strange to someone not aware of the storybook method. It is not acceptable to expect the reader to do additional reading to be able to understand the study and this paper. A brief description of the method of data collection with children would fix this, in addition the reference for additional reading.

We did briefly explain the Story Book methodology in the initial submission and hope that the addition of Supporting Information 1 provides sufficient information on this method without needing to elaborate further in the main text:

> *The SB methodology was developed by the research team to hear from children aged five to 11 years old about how an imaginary 'hero' character, approximately their age and living in one of the Cox's Bazar camps, might experience self-wetting. Children were asked to express their understanding, experiences and feelings of facing self-wetting issues through their drawings and discussions. Development of the methodology is outlined in Supporting Information 1. The SB approach was developed using a participatory process with local contextualization and is further discussed and evaluated elsewhere [21].*

We do note that we didn't elaborate on the other methods in the main text, so have now added:

> *The IDIs with caregivers and KIIs focused on understanding the experiences of children who self-wet and their caregivers, as well as providing opportunities for participants to provide suggestions on how self-wetting might be prevented. The sanitary surveys evaluated the toileting facilities used by the children in the study for their appropriateness to children of this age group, using the CHILD-SAN approach [22].*

All of the data collection tools are available online via the Open Science Framework. We decided to store all of these in one place and provide a weblink, so as to avoid having a multitude of Supporting Information files. However, we have noted that in the initial submission, the actual link did not appear. So, we have now added the link in its entirety whenever we refer to the location of the data collection tools (as well as ethical applications, participant consent forms, etc.). e.g.:

> *All of the tools used (i.e., guidance on conducting the SB approach, interview guides, sanitary surveys) are available in English and Bangla via the Open Science Framework [25] (www.doi.org/10.17605/OSF.IO/E3KHV).*

If Reviewer 2 insists, we can provide these tools as Supporting Information.

Did the data collection methods have any protocol for what to do if a child disclosed abuse or exhibited psychological distress? Was there any protocol to refer to child protection services? Did sessions continue when children exhibited distress? Was a psychologist available?

Yes, and we have added this in the manuscript:

> *A process was in place to refer children to child protection services in the camps if any indicated psychological distress.*

The first statement of the results is a sweeping generalization which is not supported by the study design. The authors attribute the findings of a single study in Cox's Bazar to all humanitarian settings.

We have altered the sentence to be clearer that we are only reporting findings from a particular context:

> *"The SB sessions, IDIs, KIIs and sanitation survey provided insight into three major facets of self-wetting by children in this humanitarian context."*

What is a Hakeem? This is referred to in a couple of places but never explained.

We have added an explanation:

"Those caregivers who consider it a disease do so because children urinate in their sleep on a daily basis and continue to do so even after consulting with doctors or Hakeem (a herbal medicine practitioner, specially of Unani medicine)."

Page 12 line 279- typos: the family size is presented as a fraction rather than a range.

This has been corrected.

Page 13 lines 289-291 – the authors make inferences which do not appear to be based on objective findings from the study (e.g. "seem…")

We use the term 'seemed' here because, as described in the Methods, we did not ask children to talk about their own experiences, but those of their 'hero', who was imaginary. From the language used and drawings the children provided, as well as the interviews with caregivers, it appeared that in some cases children were referring to their own experiences. We hope this is clearer to Reviewer 2 now that we have included more information on the SB approach (Supporting Information 1)

There is a lot of repetition of the findings in the discussion section. The discussion should not repeat the results section but should explore the meaning of the findings and how this compares to what else is known on the topic.

We have significantly reduced the Discussion section to remove replication of the Results.

The conclusion could be much stronger - could improving the toilets do more than improve the lives of children who self-wet? What about the driving factors – and likely impacts on a much larger population? And how would improving the toilets improve other aspects of the children's and families' lives?

We have re-written the Discussion to be clearer that our study could not investigate all of the driving factors of self-wetting, only those disclosed by participants (which are subjective), and that it would be useful for a future study to delve further into the myriad complexities of what causes self-wetting in such contexts;

> *"The model could likely be expanded to include more factors which contribute to negative experiences and impacts as further studies are conducted, providing further guidance on how experiences of children and caregivers could be improved."*

In response to reviewer 2's other concern, although improving the toilets would likely have a greater impact than simply improving the immediate lives of children who self-wet, and their caregivers (for example, improving accessibility for those with disabilities, allowing more individuals to attend school/work/social activities) we don't believe the Conclusion of this manuscript is the place to elaborate on the many benefits of improved WASH services in humanitarian settings. These have been discussed at length elsewhere and weren't a core focus of our data collection methods. Our methods prompted participants to think more deeply about the immediate experiences related to self-wetting rather than longer term/at larger scale. It would be interesting to hear from participants what they believe the longer term/larger scale impacts of better WASH facilities would be, but that would require a different set of research methods.
